# Previous induced abortion or miscarriage is associated with increased odds for gestational diabetes: a nationwide register-based cohort study in Finland

**DOI:** 10.1007/s00592-023-02047-6

**Published:** 2023-03-01

**Authors:** Matias Vaajala, Rasmus Liukkonen, Ville Ponkilainen, Maiju Kekki, Ville M. Mattila, Ilari Kuitunen

**Affiliations:** 1 Faculty of Medicine and Life Sciences, University of Tampere, Tampere, Finland; 2 Department of Surgery, Central Finland Central Hospital Nova, Jyväskylä, Finland; 3 Department of Obstetrics and Gynecology, Tampere University Hospital, Tampere, Finland; 4 Faculty of Medicine and Health Technology, Center for Child, Adolescent and Maternal Health Research, Tampere University, Tampere, Finland; 5 Department of Orthopaedics and Traumatology, Tampere University Hospital Tampere, Tamper, Finland; 6 Department of Pediatrics, Kuopio University Hospital, Kuopio, Finland; 7 Institute of Clinical Medicine and Department of Pediatrics, University of Eastern Finland, Kuopio, Finland

**Keywords:** Gestational diabetes, Abortion, Miscarriage

## Abstract

**Aims:**

The aim of this study was to investigate the association between previous induced abortion or miscarriage and the development of gestational diabetes mellitus (GDM) using high-quality register data.

**Methods:**

In this retrospective nationwide register-based cohort study, data from the national medical birth register (MBR) were used to evaluate the association between a history of miscarriage or induced abortion and GDM. We included all first pregnancies ending in delivery in which the oral glucose tolerance test was performed between 2004 and 2018. A logistic regression model was used to assess the development of GDM in the first pregnancy ending in delivery. Adjusted odds ratios (aOR) with 95% confidence intervals (Cis) were compared between groups.

**Results:**

In total, 15,873 nulliparous women with a history of induced abortions, 22,337 with a history of miscarriages and 3594 with a history of both were found. The reference group consisted of 138,869 women without a history of induced abortions or miscarriages. Women with a history of induced abortions (24.7%, aOR 1.15 [CI 1.11–1.20]), a history of miscarriages (24.8%, aOR 1.14 [CI 1.10–1.18]) and a history of both (27.7% aOR 1.18 [CI 1.09–1.28]) had higher odds for the development of GDM when compared to the reference group (20.8%). The odds for GDM increased along with the increasing number of previous induced abortions and miscarriages.

**Conclusion:**

Women with a history of induced abortions or miscarriages had higher odds for GDM in their first pregnancy leading to birth. Knowledge of this association will be helpful in the prevention and screening of GDM.

**Supplementary Information:**

The online version contains supplementary material available at 10.1007/s00592-023-02047-6.

## Aims

Induced abortions and miscarriages are common events worldwide. It is estimated that up to 30% of all pregnancies worldwide are terminated by induced abortion [1]. A few studies have been recently published that investigate the association between a history of pregnancy termination and subsequent risk for the development of gestational diabetes (GDM). In these studies, a history of terminated pregnancies was found to be associated with an increased risk for gestational diabetes (GDM). However, as this topic is relatively new, the evidence to support this finding is lacking in the literature and needs clarifying.

In a recent study published in 2022, the association between a history of spontaneous or induced abortion (IA) and subsequent risk of GDM was investigated. With a study population of 102,259 women, the study found that pregnant women who only experienced spontaneous abortions had a 25% higher risk of GDM, whereas women who experienced both spontaneous and induced abortions had a 15% higher risk of developing GDM. The authors of the study speculated that other conditions such as metabolic syndromes, which have been found to be associated with abortions, might partly explain this association [2]. The study concluded that further research is needed to clarify this association [2]. A recent meta-analysis study published in 2022 that focused on the association between abortions and GDM found a 41% higher risk of GDM among women with a history of recurrent abortions or miscarriages. The study also reported that the risk of GDM increased when the number of abortions increased [3]. In the meta-analysis, the authors considered the possibility that abortions could lead to increased oxidative stress, inflammation and endothelial dysfunction, which might, in turn, lead to insulin resistance and GDM [3].

Based on the hypothesis that miscarriages and induced abortions might increase the risk for the development of GDM, the aim of this study was to investigate the association between previous miscarriages and induced abortions, or both, and the development of GDM using high-quality nationwide register data.

## Materials and methods

In this retrospective nationwide register-based cohort study, data from the National Medical Birth Register (MBR), which is maintained by the Finnish Institute for Health and Welfare, were used to evaluate the association between a history of terminated pregnancies due to miscarriages or induced abortions and gestational diabetes. The MBR has high quality and coverage, the current coverage being nearly 100%. The study period was from 1 January 2004 to 31 December 2018.

**Fig. 1:**
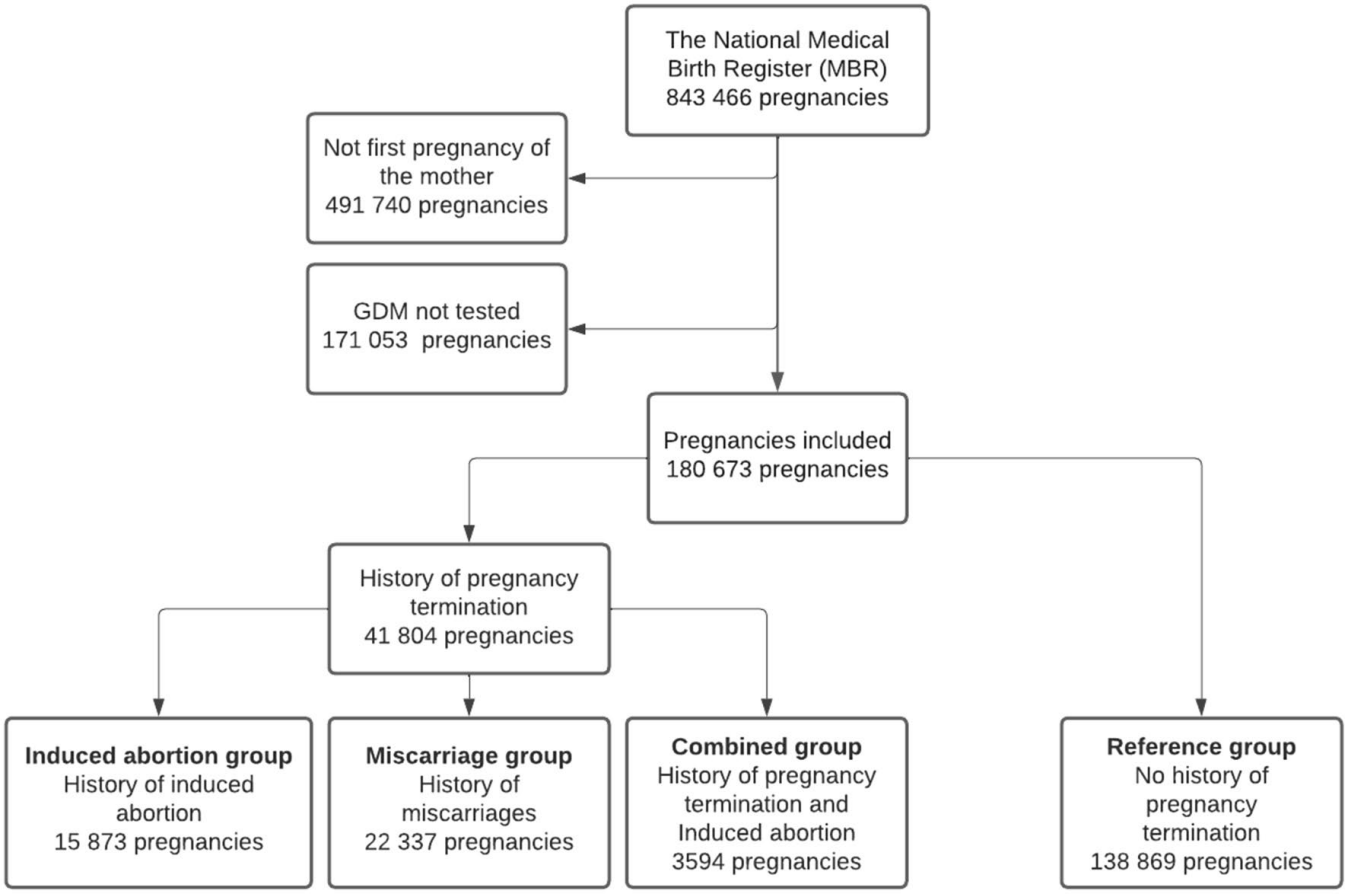
Flowchart of the study group formation. Women with a history of induced abortions, miscarriages and both (combined group) were compared to women without a history of terminated pregnancies (reference group). GDM = gestational diabetes

The MBR contains data on pregnancies, delivery statistics and the perinatal outcomes of all births with a birthweight ≥ 500 g or a gestational age ≥ 22^+0^ weeks, including information on pregnancy terminations. The number of previous miscarriages and induced abortions is recorded in the register during every pregnancy. GDM was diagnosed using the 75 g 2-h oral glucose tolerance test (OGTT). We included all first pregnancies in which an OGTT was administered and recorded in the MBR between 2004 and 2018. Only nulliparous women were included to make the groups as similar as possible and to keep the study design simple enough. In total, 180,673 pregnancies fulfilled the inclusion criteria. The patient groups with a history of recurrent pregnancy terminations were divided into three groups: 1) women with a history of IAs, 2) women with a history of miscarriages and 3) women with a history of both. In the IA and miscarriage groups, only women with IA or miscarriage were included, meaning that there were no women in these groups with a history of both IAs and miscarriages. The reference group consisted of women without previous induced abortions or miscarriages. The forming of the study cohorts is shown as a flowchart in Fig. 1.

## Statistics

The continuous variables were interpreted as means with standard deviations or as medians with interquartile ranges based on the distribution of the data. The categorical variables are presented as absolute numbers and percentages. Rates are presented with 95% confidence intervals (CIs). The CIs are calculated using Poisson regression. A logistic regression model was used to assess the primary outcome, which was the development of GDM in the first pregnancy leading to birth. The exposure variable was the history of terminated pregnancies, which included IAs, miscarriages or both. The odds for GDM were analysed separately for women who had one, two or three or more induced abortions/miscarriages. In the combined group, the odds for GDM were analysed separately for two and three or more pregnancy terminations (the sum of previous induced abortions and miscarriages). Adjusted odds ratios (aOR) with 95% CIs were compared between the groups. The model was adjusted for covariates that were found to be risk factors for GDM based on the previous studies. The selected covariates included the following: maternal age, categorized maternal body mass index (BMI) using the WHO classification, maternal smoking status, IVF [4] and multiple pregnancies [5].

The Ethical Committee of Tampere University Hospital has waived the requirement for ethical committee evaluation of all retrospective studies using routinely collected healthcare data. This decision is based on the Medical Research Act (488/1999) and the Act on the Status and Rights of Patients (785/1992). Moreover, in accordance with Finnish legislation (the Act on the Secondary Use of Health and Social Data (552/2019)), no ethical informed written consent was required due to the retrospective register-based study design and because the patients were not contacted. Permission for the use of this data was granted by Findata after evaluation of the study protocol (Permission number: THL/1756/14.02.00/2020).

## Results

In total, 15,873 nulliparous women with a history of IAs, 22,337 with a history of miscarriages and 3594 with a history of both were found in the MBR. The reference group consisted of 138,869 women without a history of IAs or miscarriages. Women were older in the miscarriage group (mean 30.2, SD 5.5) and the combined group (mean 30.6, SD 5.9) when compared to women in the reference group (mean 28.8, SD 5.0). A higher rate of smokers was found in the group of women with a history of IAs (32.1%, CI 31.2–33.0) and among women with a history of both (29.2%, CI 27.4–31.0) when compared to women in the reference group (14.4%, CI 14.2–14.6). Women in all patient groups had higher mean BMI than women in the reference group (Supplementary Table 1).

Women with a history of IAs (24.7%, aOR 1.15 [CI 1.11–1.20]), a history of miscarriages (24.8%, aOR 1.14 [CI 1.10–1.18]) and a history of both (27.7%, aOR 1.18 [CI 1.09–1.28]) had higher odds for the development of GDM when compared to the reference group (20.8%). The odds for GDM also increased along with an increasing number of IAs and miscarriages, as aOR increased by up to 1.48 (CI 1.21–1.81) after three induced abortions and by up to 1.40 (CI 1.13–1.72) after three or more IAs or miscarriages in the combined group. However, a similar increase was not found after recurrent miscarriages (aOR 1.19, CI 1.04–1.36) in the miscarriage group (Table 1).

**Table 1:** Adjusted odds ratios (aOR) with 95% confidence intervals (CI) using the logistic regression model

Logistic regression model for the development of GDM					
	Patient group				
	Number of patients GDM				
	*n*	*n*	%	aOR	CI
*Induced abortion group*					
All induced abortions	15,873	3928	24.7	1.15	1.11–1.20
One induced abortion	13,207	3180	24.1	1.12	1.07–1.17
Two induced abortions	2170	594	27.4	1.29	1.17–1.42
Three or more induced abortions	496	154	31.0	1.48	1.21–1.81
*Miscarriage group*					
All miscarriages	22,337	5544	24.8	1.14	1.10–1.18
One miscarriage	17,688	4296	24.3	1.13	1.05–1.17
Two miscarriages	3492	925	26.5	1.18	1.09–1.28
Three or more miscarriages	1157	323	28.0	1.19	1.04–1.36
*Combined group*					
In total	3594	995	27.7	1.18	1.09–1.28
Two pregnancy terminations*	2294	622	27.1	1.18	1.07–1.30
Three or more pregnancy terminations*	1300	373	28.7	1.19	1.04–1.34

*Pregnancy terminations = induced abortions or miscarriages

Women with induced abortions (IA), miscarriages or both were compared to a reference group consisting of women without previous IA or miscarriage. The model was adjusted for maternal age, categorized maternal body mass index (BMI), maternal smoking status, *in vitro* fertilization (IVF) and the number of multiple pregnancies. The reference group consisted of 138,860 pregnancies. Of these, 28,876 (20.8%) had diagnosed GDM

## Discussion

The main finding of the present study is that women with a history of induced abortions, miscarriages or both had higher odds for GDM in their first pregnancy ending in delivery. Interestingly, the odds appear to increase as the number of previous induced abortions or miscarriages increases.

In the previous literature, a prior spontaneous or induced abortion was found to be a risk factor for the development of GDM [2, 3]. The most important studies investigating this association included a retrospective cohort study with a study population of approximately 100,000 women [2] and a meta-analysis [3], both of which were published in 2022. In both studies, the risk for GDM was found to be higher after induced or spontaneous abortions. Moreover, the authors of both studies concluded that more studies should be performed to clarify this association and to subsequently help prevent the development of GDM [2, 3]. As the results of the present study are in agreement with the results of the previous studies, our results serve to clarify the association between a history of induced abortions or miscarriages and the development of GDM. Moreover, the study sample in our study was nearly twofold larger than that of the previous retrospective cohort study [2]. One of the major drawbacks with the cohorts included in a meta-analysis is that they most likely have high heterogeneity, which may cause bias. Because induced or spontaneous abortions are common adverse pregnancy events [1], knowing that they are a possible risk factor for GDM and using this knowledge for the prevention of GDM is a truly important step.

Although the biological mechanisms between abortion and GDM are not fully understood, the previous studies have speculated that metabolic syndromes in the background, increased oxidative stress, inflammation or the endothelial dysfunction caused by abortions might lead to GDM in later pregnancies [2, 3]. Additionally, GDM might have already been present in those pregnancies that ended in spontaneous or induced abortion. Other lifestyle differences, which are not measurable in our data, might also explain the association. However, based on our data, the exact reason remains unknown. Therefore, as long as the biological mechanisms between abortions and GDM remain unknown, knowledge of the association should be used in the screening and prevention of GDM. Thus, women with a history of miscarriages or induced abortions should be asked to attend more frequent antenatal visits to monitor their blood glucose and implement early prevention.

The strengths of our study are the large amount of nationwide register data used and the long study period. Compared to a previous retrospective cohort study using a study population of approximately 100,000 women, our study sample was nearly twofold higher. The register data used in our study are routinely collected in structured forms using national instructions, which ensures good coverage (over 99%) and reduces possible reporting and selection biases. As the number of women in the study cohorts was large, it was also possible to analyse the effects of multiple pregnancy terminations on the odds for GDM. A possible limitation of this study is that after 2008 the screening methods for GDM changed to comprehensive screening, meaning that GDM testing rates increased towards the end of the study period.

## Conclusions

The main finding of this study is that women with a history of induced abortions, miscarriages or both had higher odds for GDM in their first pregnancy leading to birth. The odds also appear to increase as the number of previous induced abortions increases. Therefore, a history of previous induced abortion or miscarriage should be acknowledged as a possible risk factor for the development of GDM, and the results gained from this study should be used in the prevention and screening of GDM.

## Supplementary Information

Below is the link to the electronic supplementary material.

Supplementary file1 (PDF 48 KB)
