# Supplementary material for: Previous induced abortion or miscarriage is associated with increased odds for gestational diabetes: a nationwide register-based cohort study in Finland
Source: Acta Diabetol. 2023 Mar 1;60(6):845–9. doi: 10.1007/s00592-023-02047-6 (PMC10148769; doi:10.1007/s00592-023-02047-6)
Supplement: Supplementary file 1 — Supplementary file1 (PDF 48 KB) [file 592_2023_2047_MOESM1_ESM.pdf]

Supplementary table 1: Background information on the study groups

	**Induced abortion group**		**Miscarriage group**		**Combined [illegible]**	
Total number of patients	15 873		22 337		3594[illegible]	
	**n**	**% (CI)**	**n**	**% (CI)**	**n**	[illegible]
Age (mean; sd)	28.6 (5.3)		30.2 (5.5)		30.6 (5.9)	
Smoking status smoker	5096	32.1 (31.2-33.0)	3222	14.4 (13.9-14.9)	1048	2[illegible]
BMI (Kg/m^2) (mean; sd)	25.9 (5.2)		25.7 (5.3)		26.2 (5.5)	
underweight (BMI < 18.5)	364	2.3 (2.1-2.5)	419	1.9 (1.7-2.1)	60	[illegible]
normal weight (18.5 ≤ BMI < 25.0)	7588	47.8 (46.7-48.9)	11 132	49.8 (48.9-50.8)	1677	4[illegible]
overweight (18.5 ≤ BMI < 25.0)	4837	30.5 (29.6-31.3)	6461	28.9 (28.2-30.0)	1084	3[illegible]
obesity class I (25.0 ≤ BMI < 30.0)	1938	12.2 (11.7-12.8)	2723	12.2 (11.7-12.7)	488	1[illegible]
obesity class II (18.5 ≤ BMI < 40.0)	742	4.7 (4.3-5.0)	936	4.2 (3.9-4.5)	171	[illegible]
obesity class III (40.0 ≤ BMI)	269	1.7 (1.5-1.9)	440	2.0 (1.8-2.2)	88	[illegible]
BMI missing	134	0.8 (0.7-1.0)	226	1.0 (0.9-1.2)	26	[illegible]
Multiple pregnancy	207	1.3 (1.1-1.5)	445	2.0 (1.8-2.2)	65	[illegible]
Pregnancy started with IVF	27	0.2 (0.1-0.2)	144	0.6 (0.5-0.8)	6	[illegible]
